# Circulating D-dimer level correlates with disease characteristics in hepatoblastoma patients

**DOI:** 10.1097/MD.0000000000008798

**Published:** 2017-11-27

**Authors:** BinBin Zhang, GongBao Liu, XiangQi Liu, Shan Zheng, Kuiran Dong, Rui Dong

**Affiliations:** a Children's Hospital of Fudan University; b Department of Pediatric Hepatobiliary Surgery, Children's Hospital of Fudan University; c Key Laboratory of Neonatal Disease, Ministry of Health, Shanghai, China.

**Keywords:** D-dimer, hepatoblastoma, pediatric tumor, tumor biomarker

## Abstract

**Objectives:**

Hepatoblastoma (HB) is the most common pediatric liver malignancy, typically affecting children within the first 4 years of life. However, only a few validated blood biomarkers are used in clinical evaluation. The current study explored the clinical application and significance of D-dimer levels in patients with HB.

**Method:**

Forty-four patients with HB were included in this retrospective study. D-dimer and plasma fibrinogen levels were examined, and their correlation with other clinical features was analyzed. D-dimer and plasma fibrinogen levels were examined between HB and other benign hepatic tumors.

**Results:**

D-dimer levels were significantly associated with high-risk HB features, such as advanced stage and high α-fetoprotein (AFP) levels. Higher D-dimer levels were observed in stage 4 patients compared with stage 1/2/3 patients (*P* = .028). Higher D-dimer levels were also observed in patients with higher AFP levels before chemotherapy compared with patients with lower AFP levels after chemotherapy (*P*< 0.001). In addition, higher D-dimer levels were observed in HB compared with other benign hepatic tumors such as hepatic hemangioma and hepatocellular adenoma (*P* = .012). No significant difference in D-dimer levels was found between sex (*P* = .503) and age (≥12 vs <12 months, *P* = .424). There was no significant difference in plasma fibrinogen levels between sex or age and high-risk HB features, such as advanced stage and high AFP levels.

**Conclusions:**

Elevated D-dimer levels could be a useful determinant biomarker for high-risk features in patients with HB. This finding also supports the clinical application of D-dimer in HB.

## 1 Introduction

Although rare in adults, hepatoblastoma (HB) is a common liver malignancy in children, and it has the third highest incidence among abdominal solid tumors, after neuroblastoma and Wilms tumor.^[1,2]^ HB is the most common form of childhood liver cancer, accounting for approximately 80% of liver cancers and 1% of malignant tumors in children, and it affects 0.5 to 1.5 per million children per year.^[3,4]^ This disease is often observed in boys between the ages of 6 months and 4 years, and it is rarely diagnosed in adults.^[5,6]^ One review showed that 67% of HBs were epithelial, with a combination of mixed embryonal and fetal patterns; 21% displayed a mesenchymal component in addition to the common epithelial patterns. Approximately 7% of the total were composed of a pure, well differentiated fetal epithelial component, and 5% demonstrated primitive-appearing or small cell undifferentiated tumor cells.^[5,6]^

HB is composed of malignant tumor cells derived from differentiated and proliferating pluripotent stem cells during embryonic development.^[7,8]^ It is characterized by heterogeneous tumors and a broad spectrum of clinical behaviors, some of which completely regress spontaneously, whereas others proliferate and progress. Surgical removal of the tumor, adjuvant chemotherapy, and liver transplantation have been used to treat these cancers.^[9]^ HB is a curable malignancy in children and early diagnosis and therapy are effective at increasing survival rates.^[10]^ However, this type of cancer has no specific clinical manifestations. Most children are admitted to the hospital because of a large abdominal mass and they already have stage 2 or higher disease; some patients even have metastases to the hilus hepatis, portal vein, and brain at diagnosis.^[11]^ Therefore, clarifying the disease extent and stage at the time of diagnosis can improve the survival rate of children with HB.^[10]^ Blood biomarkers have been widely applied in clinical laboratory examinations, serving as references for diagnosis, disease monitoring, and prognosis prediction. However, only a few validated blood biomarkers have been used in HB; the most common method of testing for HB is a blood test that assesses the alpha-fetoprotein (AFP) level. The highest levels of the glycoprotein AFP produced by endodermal tissues are physiologically seen during fetal development, where approximately 90% of children with HB have elevated serum AFP levels at diagnosis.^[8,12,13]^ However, some patients with HB with negative AFP levels show an advanced disease stage at the point of diagnosis, and they also show chemoresistance and have a poor outcome.^[14]^ Thus, development of suitable biomarkers for HB evaluation remains an unmet clinical need.

Activation of coagulation and fibrinolysis has been found to be frequently associated with malignancies and involved in angiogenesis, tumor cell invasion, tumor progression, and prognosis.^[15]^ Cross-linked fibrin in the extracellular matrix serves as a stable framework for endothelial cell migration during angiogenesis and tumor cell migration during invasion.^[16]^

D-dimer, a fibrin degradation product, is elevated by increasing fibrin formation and fibrinolysis.^[17]^ Clinically, it has been widely used in the assessment of potential thrombotic episodes, such as in suspected acute venous thromboembolism. An elevated D-dimer level is also observed in cancer patients, which indicates the occurrence of venous thromboembolism. The relationship between D-dimer and carcinoma has been reported in several solid tumors, such as lung cancer, renal cell carcinoma, and colorectal cancer.^[18–22]^ However, little information is known about D-dimer in HB.

Plasma fibrinogen is an important component in the coagulation pathway. High levels of serum fibrinogen may be associated with increased fibrinogen deposits in tumor tissue and serve as an extracellular matrix for tumor cell adhesion or migration, which may result in tumor metastasis, promote tumor neovascularization and angiogenesis, enhance adhesion and invasion, and play an important role in cancer progression.^[23]^ The relationship between fibrinogen and carcinoma has been reported in several solid tumors, such as renal cell carcinoma, resectable pancreatic carcinoma, human nasopharyngeal carcinoma, upper urinary tract urothelial carcinoma, and hepatocellular carcinoma.^[23–27]^

In the present study, data from 44 children who were diagnosed with HB at Children's Hospital Fudan University (Shanghai, China) between July 2013 and March 2017 were retrospectively analyzed. All of the HB patients had no other diseases that influenced D-dimer or plasma fibrinogen levels. The correlation of D-dimer and plasma fibrinogen levels with clinical features as well as the prognostic value was investigated. The outcomes of the study might indicate a novel blood biomarker for clinical evaluation of HB patients.

## 2 Materials and methods

### 2.1 Patients and disease characteristics

Medical records for all patients who were diagnosed with HB between July 2013 and March 2017 were reviewed retrospectively. Forty-four patients with complete records were included in the study. Because the D-dimer level and plasma fibrinogen is frequently influenced in many diseases such as deep vein thrombosis, all patients with HB enrolled into this study had neither of these diseases. Among them, 30 patients (17 boys and 13 girls) underwent at least 2 rounds of chemotherapy (cisplatin + vincristine + 5-fluorouracil). AFP levels in 30 patients declined noticeably after chemotherapy. All patients were followed up until March 2017. All of the medical records were reviewed after obtaining written informed consent. The Ethics Committee of the Children's Hospital of Fudan University approved the use human blood samples.

Disease extent and diagnosis were assessed using computerized tomography (CT), magnetic resonance imaging (MRI), B-scan ultrasonography, or bone scans. AFP levels were also analyzed. Disease staging was evaluated according to the PRE-Treatment tumor EXTension (PRETEXT) guidelines. The PRETEXT system was developed by the International Childhood Liver Tumours Strategy Group (SIOPEL) and is based on Couinaud's classification of the liver into 8 segments.^[28]^ These segments are further grouped into 4 sections: left lateral, left medial, right anterior, and right posterior. PRETEXT staging is determined by the number of adjoining sections that are free of tumor: PRETEXT I, 3 adjoining sections are free of tumor; PRETEXT II, 2 adjoining sections are free of tumor; PRETEXT III, no 2 adjoining sections are free of tumor; and PRETEXT IV, all sections are involved.

Measurements of D-dimer were made using a turbidimetric inhibition immunoassay at our hospital. Measurements of plasma fibrinogen were made using a Clauss method at our hospital, and they were included in the evaluation at diagnosis. The plasma D-dimer normal level at our hospital is <0.3 mg/L and plasma fibrinogen normal level at our hospital is 2 to 4 g/L.

### 2.2 Statistical analysis

Statistical analyses and graphical depiction of data were generated using GraphPad Prism 6.0 (GraphPad Software, Inc., La Jolla, CA). Graphical depiction of data was completed using SPSS 19.0 software (IBM SPSS, Armonk, NY). The D-dimer/fibrinogen values before and after chemotherapy were analyzed using a paired sample *t* test (2-tailed). The other measurement data (sex, age, stage, malignant, and benign) were analyzed using an independent sample *t* test. *P* < 0.05 was considered to indicate a statistically significant difference.

## 3 Results

### 3.1 Patient characteristics

Forty-four HB patients (25 boys and 19 girls) with complete medical records and information were included in the study. Table 1 shows the clinical and biological characteristics of HB patients. Table 2 shows the clinical and biological characteristics of hepatic benign tumor patients. Table 3 shows the pre/postchemotherapy AFP levels of 30 patients. The median D-dimer level was 2.30 mg/L.

**Table 1:**
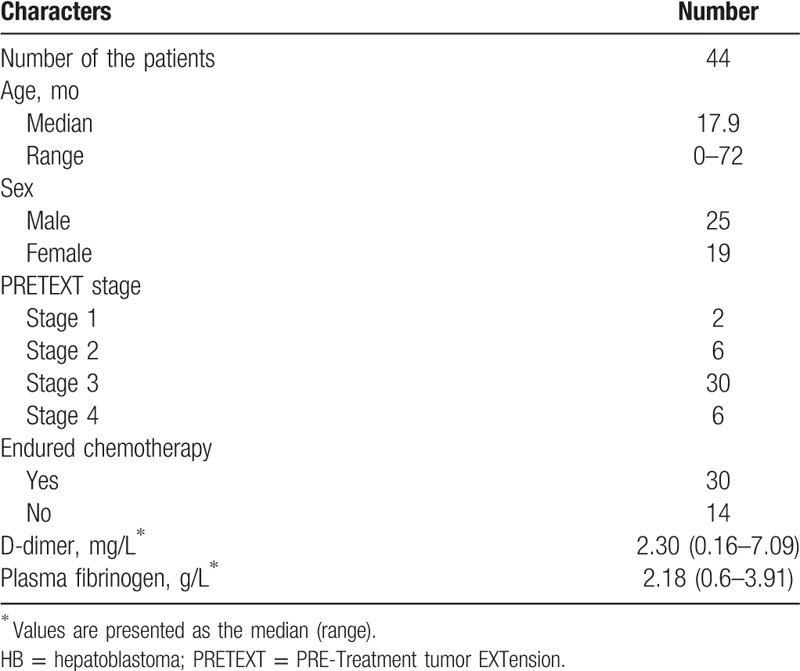
Clinical characteristics of patients with hepatoblastoma.

**Table 2:**
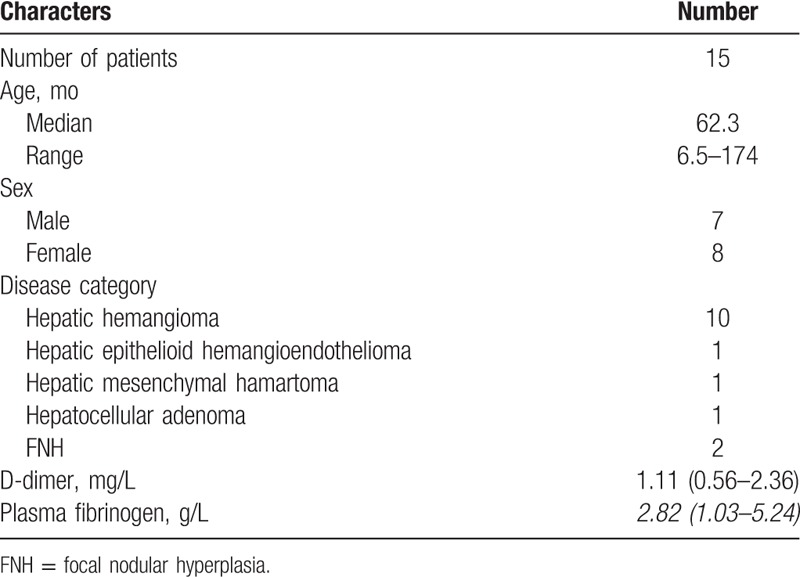
Clinical characteristics of patients with hepatic benign tumor.

**Table 3:**
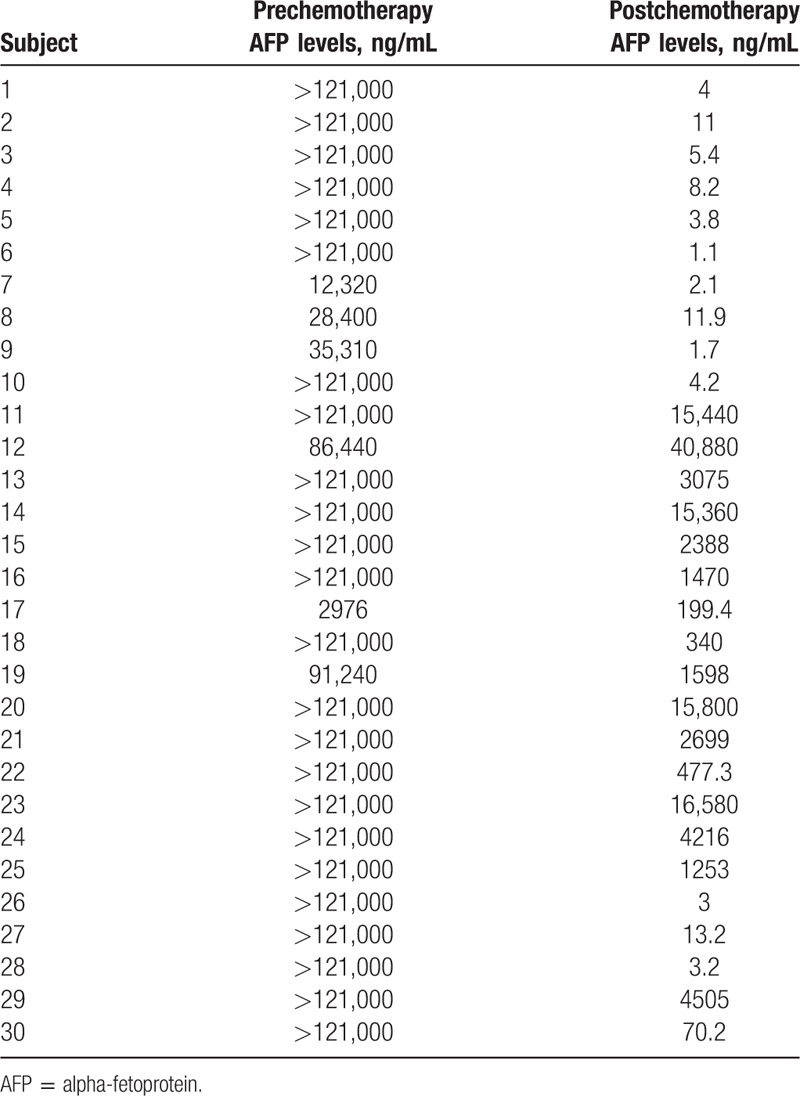
Pre/postchemotherapy alpha-fetoprotein levels in 30 patients.

### 3.2 D-dimer level subgroup analysis

**Figure 1:**
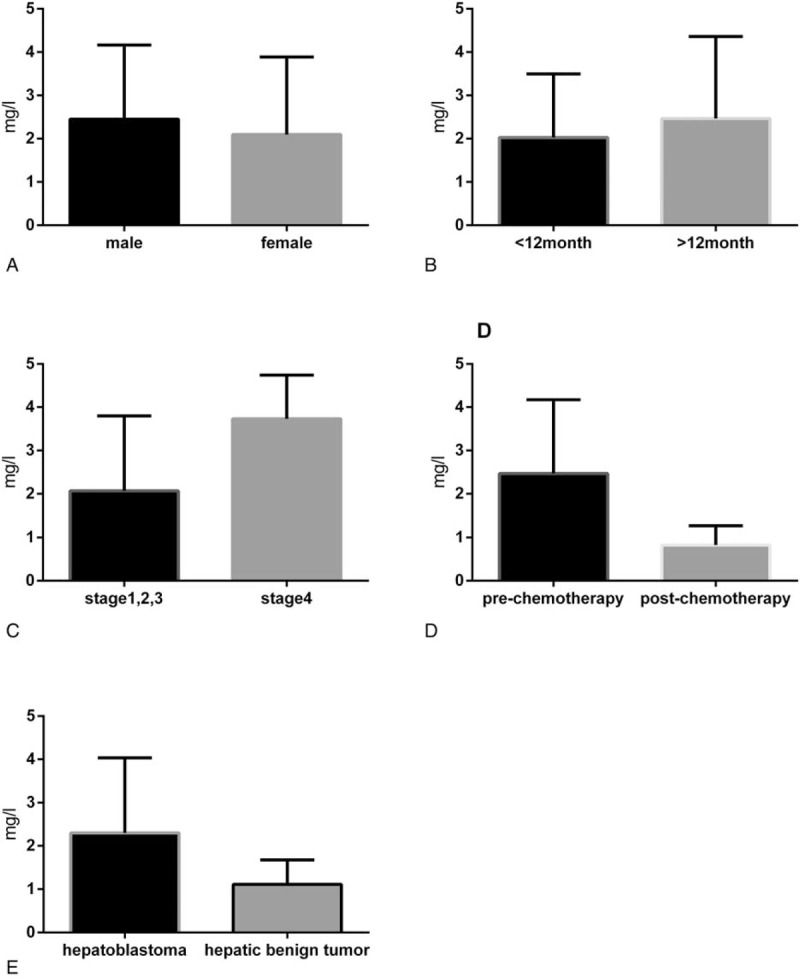
Association between D-dimer levels and clinicopathologic features. A, Sex: male versus female (*P* = .503). B, Age: ≥12 months versus <12 months (*P* = .424). C, Disease stage: stage 1/2/3 versus stage 4 (*P* = .028). D, Prechemotherapy versus postchemotherapy (*P* < .001). E, Hepatoblastoma versus hepatic benign tumor (*P* = .012).

As shown in Figure 1, subgroup analysis of D-dimer levels was performed. There was no significant difference in D-dimer levels between men and women (*P* *=* .503). Similarly, no significant difference was observed between age groups (≥12 vs <12 months, *P* *=* .424), although there were higher mean levels of D-dimer in the ≥12-month age group. Higher levels of D-dimer were found in patients with stage 4 HB compared with stage 1/2/3 HB (*P* = .028). Similarly, D-dimer levels were decreased postchemotherapy compared with prechemotherapy (*P* < .001). We also observed that the D-dimer levels in patients with HB were higher than that in patients with hepatic benign tumors, such as hepatic hemangioma and hepatocellular adenoma (*P* = .012). These results suggest that D-dimer is associated with high-risk features, such as advanced stage and malignancy.

### 3.3 Plasma fibrinogen level subgroup analysis

**Figure 2:**
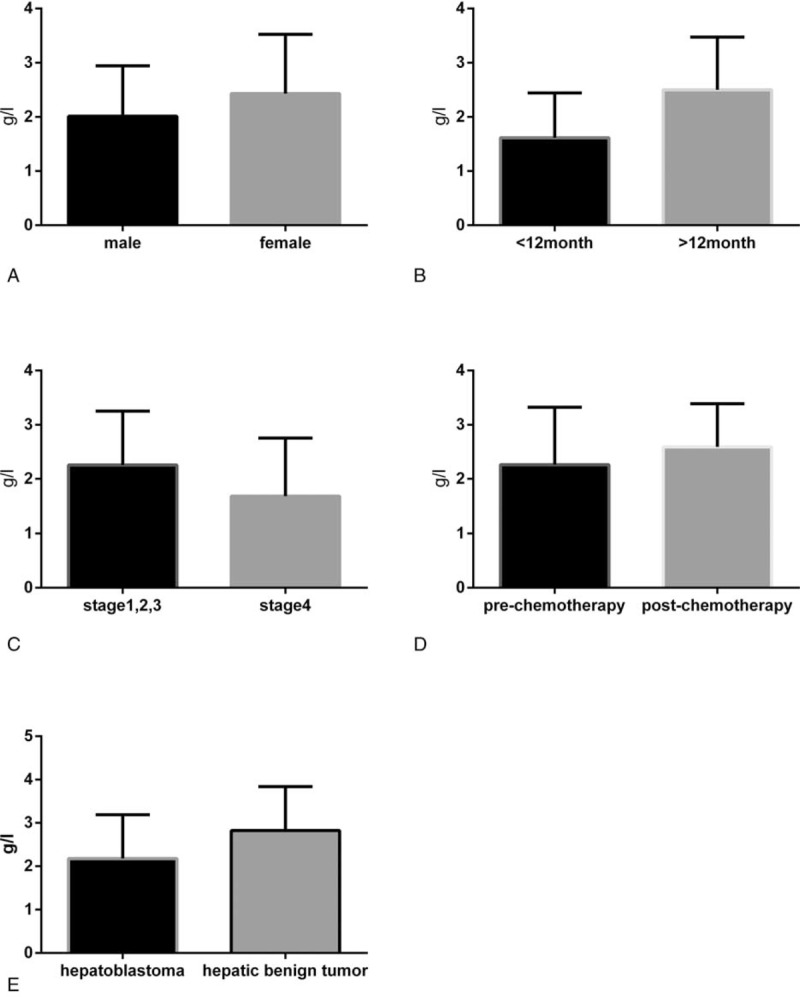
Association between plasma fibrinogen levels and clinicopathologic features. A, Sex: male versus female (*P* = .587). B, Age: ≥12 months versus <12 months (*P* = .581). C, Disease stage: stage 1/2/3 versus stage 4 (*P* = .907). D, Prechemotherapy versus postchemotherapy (*P* = .119). E, Hepatoblastoma versus hepatic benign tumor (*P* = .816).

As shown in Figure 2, no significant difference in the plasma fibrinogen levels was found between sex or age and high-risk HB features. There was also no significant differences between the following: D-dimer levels between men and women (*P* = .587); age groups (≥12 vs <12 months, *P* = .581); patients with stage 4 HB compared with stage 1/2/3 HB (*P* = .907); postchemotherapy compared with prechemotherapy (*P* = .119); and patients with HB compared with patients with hepatic benign tumors (*P* = .816).

### 3.4 Survival according to D-dimer at diagnosis

In the present study, all children were followed up for at least 2 years after being diagnosed. We did not analyze overall survival in the present study because all children survived during the limited follow-up duration.

## 4 Discussion

The current staging system and risk stratification system rely on conventional diagnostic modalities such as CT, MRI, and various radioisotope scans, and clinical characteristics such as age and sex. Patients are stratified into risk groups based on these staging systems. Patients within each risk group, however, still showed varying outcomes and responses to the same treatment. Improving the staging and risk stratification system may be considered by adding new parameters. Tumor markers were used in the initial assessment of patients with HB. AFP levels at diagnosis have been reported to be a prognostic factor, with an initial AFP level <100 or >1,000,000 ng/mL associated with worse outcomes. Some patients with HB with negative AFP levels show an advanced disease stage at the point of diagnosis, and they also show chemoresistance and have a poor outcome. Thus, development of suitable biomarkers for HB evaluation remains an unmet clinical need.

In our study, we found that the D-dimer level is closely associated with HB clinical features. Patients with a high risk, those who had a higher AFP level before chemotherapy, or those classified as stage 4 showed a higher D-dimer level, indicating that D-dimer is potentially a biomarker for high-risk features. This may be because of the importance of cross-linked fibrin in angiogenesis and tumor invasion within a favorable host environment. Fibrinogen is an important source of fibrin, which plays a crucial role in circulating tumor cell (CTC) extravasation and distant metastasis development.^[29]^ D-dimer, the final stable product of fibrin, which is elevated after enhanced activation of the coagulation and fibrinolysis system, is widely used to detect and exclude deep vein thrombosis and associated thromboembolic diseases. It may be associated with CTC appearance and it can reflect the metastatic phenotype of cancer patients.^[30]^ Fibrin remodeling is critical for formation of new vessels and is involved in many steps of metastasis.^[31]^ Thrombin, a central enzyme in the clotting cascade, also functions as a potent tumor promoter. Thrombin-mediated activation of platelets induces adhesion of tumor cells to platelets and the formation of a clot around tumor cells in the circulation.^[32]^ Therefore, it protects the tumor cells against immune system monitoring and attack, thus resulting in metastasis. We found that mice that were deficient in plasminogen developed larger tumors and more distant metastases, resulting in a decreased lifespan.^[33,34]^ In addition, the relationship between D-dimer levels and clinical features has been reported in other tumors. In small cell lung cancer, no significant correlation was identified between D-dimer levels and age or between sex and smoking, but D-dimer was correlated with tumor stage and the number of metastases.^[20,22]^ In colorectal cancer patients, preoperative D-dimer levels were associated with larger tumor sizes, deeper wall penetration, and tumor metastasis.^[21,35]^ Additional associations were found with pancreatic cancer, ovarian cancer, and breast cancer.^[21,35]^ All of these findings together with the present study in patients with HB suggest that D-dimer levels are associated with more aggressive disease. Thus, D-dimer may become a candidate for a staging system and risk stratification system in HB. The identification of new markers will contribute to the development of better strategies for patient management.

This study was designed to obtain knowledge, using primary information sources, about the potential clinical application of D-dimer in patients with HB, and to test its association with high-risk features. Higher D-dimer levels were observed in stage 4 patients compared with stage 1/2/3 patients. Higher D-dimer levels were observed in patients with higher AFP levels before chemotherapy compared with patients with lower AFP levels after chemotherapy. Higher D-dimer levels were also observed in HB compared with other benign hepatic tumors such as hepatic hemangioma and hepatocellular adenoma. To our knowledge, this is the first study to report that D-dimer is a useful biomarker that reflects the high-risk nature of HB.

This study has several limitations. First, the sample size is small. The prevalence rate of HB is low among the population, and although our hospital is the largest pediatric hospital in eastern China, we only receive approximately 40 newly diagnosed HB patients per year. After exclusion of ineligible patients, such as those with incomplete records or those who were lost to follow-up, we enrolled only 44 patients into the study. The small number of events will increase the bias in the analysis. Second, this is a retrospective study, rather than a prospective study. A prospective study will provide stronger evidence of D-dimer's clinical significance in HB. Third, we did not analyze overall survival in the present study because of the limited follow-up duration. Overall survival would provide more information and evidence of D-dimer's impact on the therapeutic response. Fourth, plasma fibrinogen is also an important component in the coagulation pathway, and the relationship between fibrinogen and carcinoma has been reported in several solid tumors. The explanation for no significant difference observed for plasma fibrinogen levels between sex or age and high-risk HB features is not clear. Finally, D-dimer is not a specific biomarker for HB or for tumors.

In summary, elevated D-dimer levels could predict more aggressive disease. Considering the rapidness, validity, and low expense of D-dimer testing, routine measurement of D-dimer level in HB patients can be considered in clinical practice, especially in developing countries. This could provide useful information for patient management.
